# Modern Sociology

**Published:** 1900-01-06

**Authors:** 


### Modern Sociology.

### SELFISH PARENTS—STARVING CHILDREN.

By a Correspondent.

The discovery of the large number of children in the Board schools of London who are habitually underfed has roused a strong wave of pity through the country. It is true that that pity has not always taken a wise or reasonable shape. Many have cried out, "What is the good of educating these children if they are starving?" The School Board of London has been spoken of as if it had done these children an injury in taking them from their garrets and their gutters and offering them an education which, if properly assimilated, might lead them out of garrets and gutters for ever. Perhaps, if the physical condition of the children is low, they will not assimilate the education. That is true, though not invariably true. But can a national system of education pick and choose among the children it has to do with? What would be said if the compulsory officers of the Board went about saying something equivalent to this: "You, John Smith, receive three good meals a day from your parents; therefore, you shall come to school. But you, Tom Brown, are starved and underfed; therefore, as you are hungry, the law ordains that you shall be ignorant as well. You are not to come to school, for you would get no good from the education given there." Would not this be the most gross, the most ridiculous favouritism; a *reductio ad absurdum* of the text, "To him that hath shall be given?" Yet if we may judge from some of the howls that have appeared in newspapers, this is what is desired.

But, it is said, the Board that compels these children to attend school should see to it that they are fed. To say this takes for granted two things that may reasonably be questioned; first, that there exist no charities which will undertake the duty of feeding these hungry children; and second, that all underfed children are the offspring of needy parents. Now everyone knows that in most large towns there exists a society which supplies free dinners to necessitous children, and also societies which supply penny dinners to those who do not want to accept charity, though the number of the latter is small. Do these lack funds to meet the needs of the districts they deal with? If they do, the reason is not to be sought in the meanness or indifference of the public, but in a doubt, often well founded, whether the money given is always spent judiciously. And in this doubt lies the answer to our other query. The parents of the children who ask for these free meals are not always poor. There are some who are well off, but mean. If food or anything else is to be got for nothing they will try to get a share of it. Their children will be taught to cry poverty and hunger, and to take all they can get for nothing. All charities suffer from these hypocritical pests, and many have to expend part of the money they receive for the help of the distressed in tracking out and exposing the undeserving. Before listening to the appeal for free food in addition to free education—or instead of it—we must be sure that none of this class is to share in the benefit.

There are undeniably many children who are regularly underfed because their parents are apparently in poverty. Are these all to be fed at the expense of outsiders? Before answering that we should inquire into the circumstances of the parents. To what is their poverty due? If we find a father who is earning good wages, as good as those of the man whose children are not underfed, are we justified in feeding his offspring? They are starved, not because their parents are poor, but because they are thriftless and probably vicious. If the father drinks or bets the children are almost sure to be starved and ill-clad. What are we to do in that case? Impetuous philanthropy cries out that it is not the children's fault; why should they suffer for their parents' sins? This is the cry of the heart, but that of the head must be heard. To feed the children of the drunken and degraded is only to make a present to such parents of the sum, small though it be, that under existing circumstances they are compelled to spend on their children. Underfed the children may be now; but they get some food at home. If there was a school fund to depend on for food, the home rations, poor as they are, would be stopped. Indirectly, therefore, the charity that would support the children would only encourage the drunkenness or thriftlessness of parents. And this, surely, is unjustifiable.

When cases of apparent poverty are investigated it is notable how many there are in which the excuse will not bear looking into. Take one that came up before a school board recently. A man, whose children were receiving, in addition to free education and free school books, free meals and free boots, asked if they could not also get free clothing. On inquiry it was found that the children were indeed ill-clad, but that the father was a tailor earning 35s. a week. Would pity for the children excuse the giving of free clothing to enable this man still further to escape from his parental responsibilities? In another case, where a father with the aid of his three eldest sons was earning £14 a week—that is, at the rate of £728 a year—the younger children of the family went to school with bare feet. This may be an extreme case, but less glaring ones are quite common, and those who come into closest personal contact with the poor admit the most readily that destitution does not always spring from lack of means. Even when inquiry shows that the father is out of work, it is sometimes difficult to be altogether sympathetic. He may have been thrown out of employment through his own dissipated habits, or when he could no longer get employment at the one thing he used to do he would not try any other job. This is a very common case. Another almost equally common is that of the man who will not quit London, even though he has the prospect of work elsewhere. This is why so many attempts at farm colonies have failed. And one thing is invariable, that the poverty which, whether it springs from ill-health, lack of work, or any other cause, throws the elder children on charity for food, clothing, and all else that can be had, never prevents the addition of new olive branches to the destitute family. These things may not be a reason for refusing to help the children, either by food or clothing, but they are a reason for protesting against the outcry of the amateur philanthropist, who is always ready to take the side of the poor, without asking whether they have shown reasonable prudence, industry, or respectability, such as he would expect from his own next-door neighbour. Let us help, but let us do it carefully, and with our eyes open.

